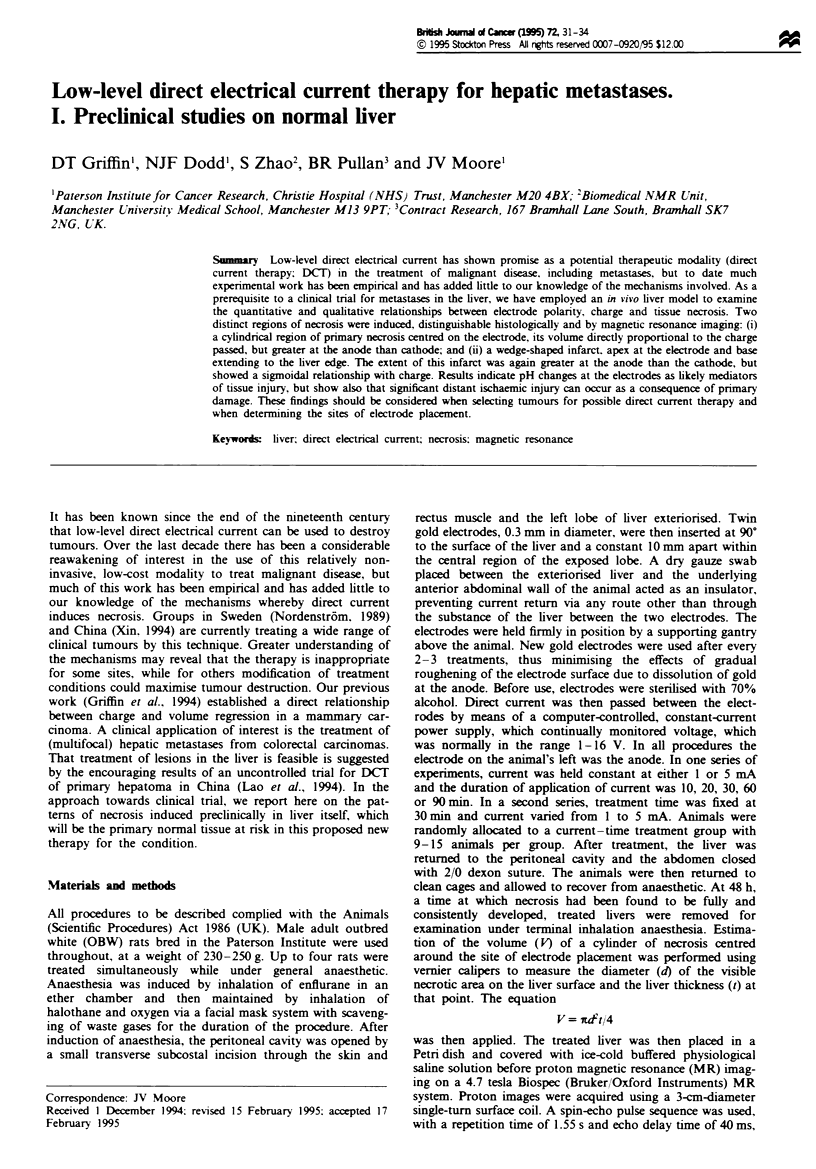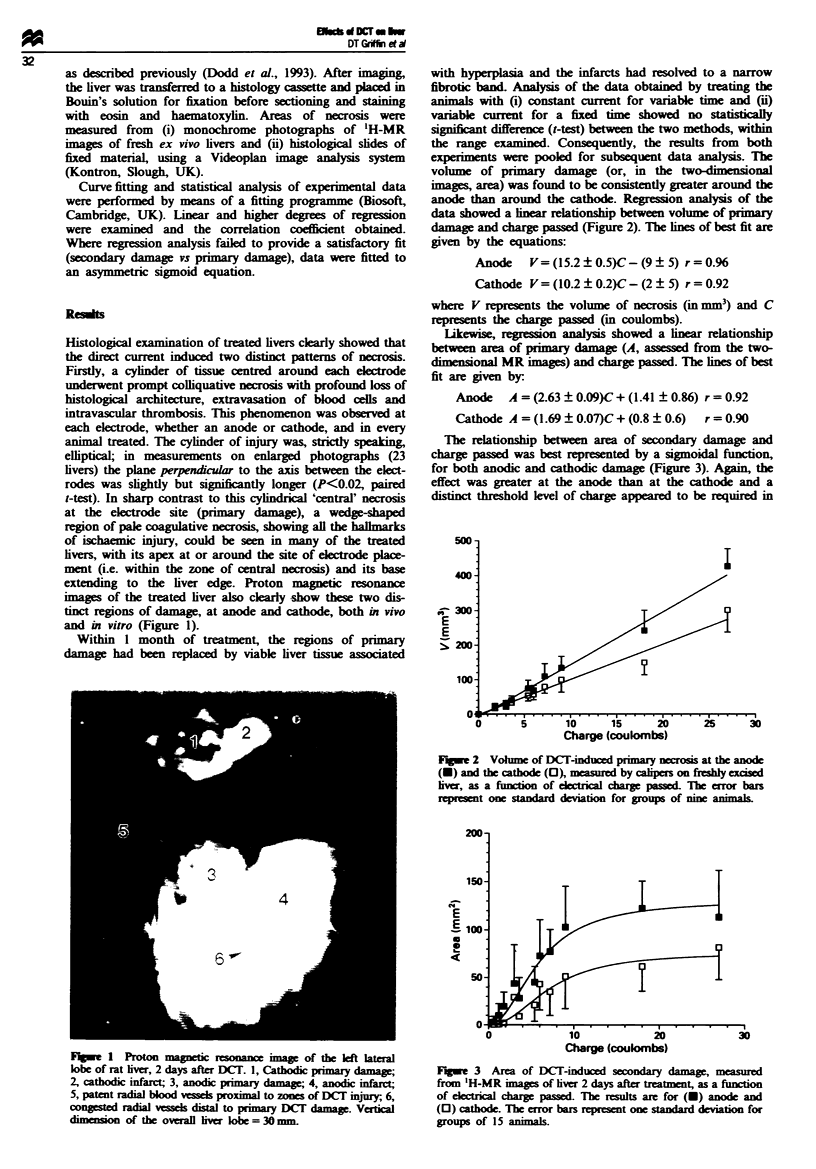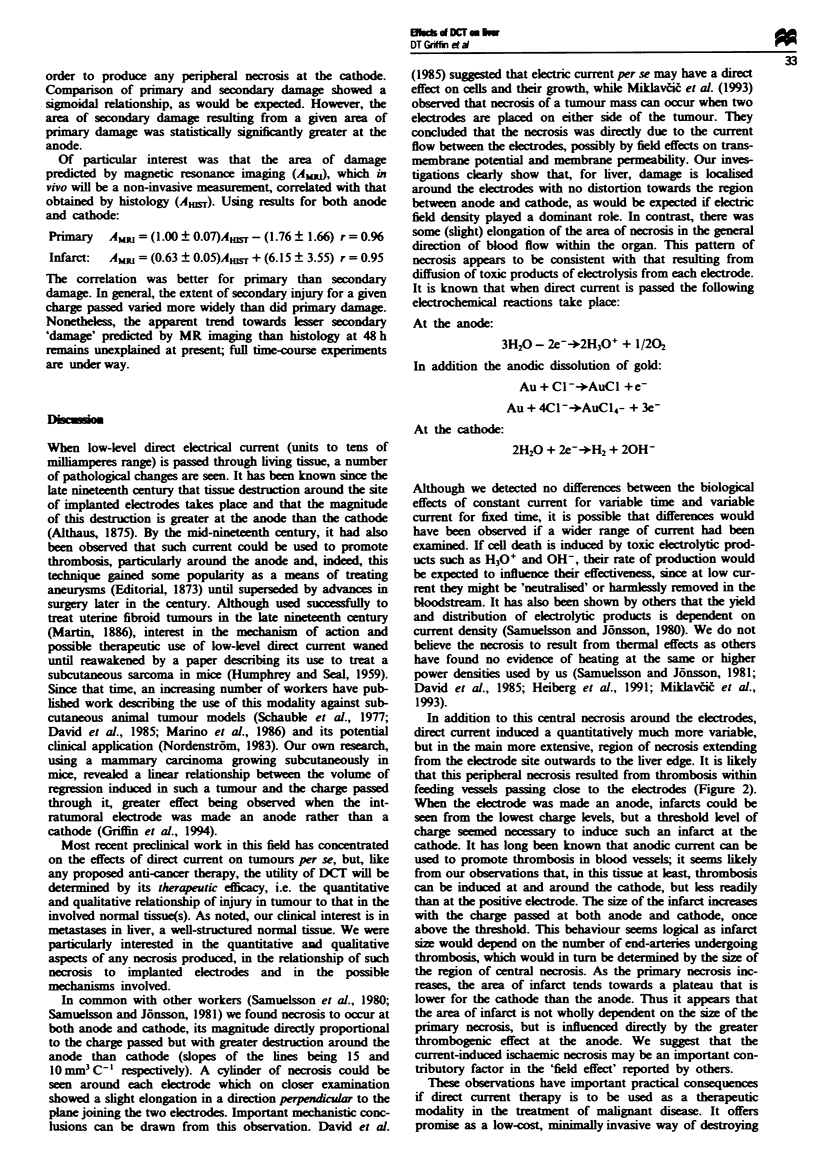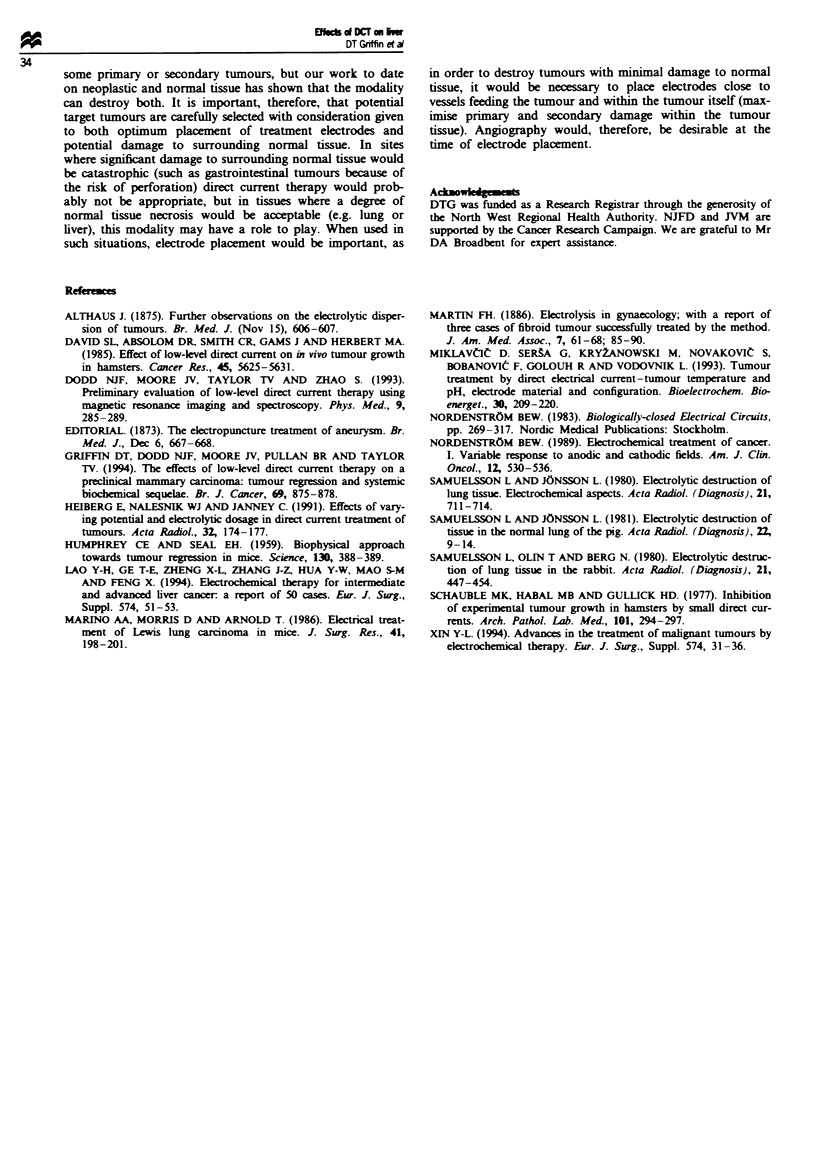# Low-level direct electrical current therapy for hepatic metastases. I. Preclinical studies on normal liver.

**DOI:** 10.1038/bjc.1995.272

**Published:** 1995-07

**Authors:** D. T. Griffin, N. J. Dodd, S. Zhao, B. R. Pullan, J. V. Moore

**Affiliations:** Paterson Institute for Cancer Research, Christie Hospital (NHS) Trust, Manchester, UK.

## Abstract

**Images:**


© 1995 Stockton Press All rights reserved 0007-0920/95 $12.00

### Low-level direct electrical current therapy for hepatic metastases. I. Preclinical studies on normal liver

DT Griffin[1], NJF Dodd[1], S Zhao[2], BR Pullan[3] and JV Moore[1]

[1]*Paterson Institute for Cancer Research, Christie Hospital (NHS) Trust, Manchester M20 4BX;* [2]*Biomedical NMR Unit, Manchester University Medical School, Manchester M13 9PT;* [3]*Contract Research, 167 Bramhall Lane South, Bramhall SK7 2NG, UK.*

**Summary** Low-level direct electrical current has shown promise as a potential therapeutic modality (direct current therapy; DCT) in the treatment of malignant disease, including metastases, but to date much experimental work has been empirical and has added little to our knowledge of the mechanisms involved. As a prerequisite to a clinical trial for metastases in the liver, we have employed an *in vivo* liver model to examine the quantitative and qualitative relationships between electrode polarity, charge and tissue necrosis. Two distinct regions of necrosis were induced, distinguishable histologically and by magnetic resonance imaging: (i) a cylindrical region of primary necrosis centred on the electrode, its volume directly proportional to the charge passed, but greater at the anode than cathode; and (ii) a wedge-shaped infarct, apex at the electrode and base extending to the liver edge. The extent of this infarct was again greater at the anode than the cathode, but showed a sigmoidal relationship with charge. Results indicate pH changes at the electrodes as likely mediators of tissue injury, but show also that significant distant ischaemic injury can occur as a consequence of primary damage. These findings should be considered when selecting tumours for possible direct current therapy and when determining the sites of electrode placement.

**Keywords:** liver; direct electrical current; necrosis; magnetic resonance

It has been known since the end of the nineteenth century that low-level direct electrical current can be used to destroy tumours. Over the last decade there has been a considerable reawakening of interest in the use of this relatively non-invasive, low-cost modality to treat malignant disease, but much of this work has been empirical and has added little to our knowledge of the mechanisms whereby direct current induces necrosis. Groups in Sweden (Nordenström, 1989) and China (Xin, 1994) are currently treating a wide range of clinical tumours by this technique. Greater understanding of the mechanisms may reveal that the therapy is inappropriate for some sites, while for others modification of treatment conditions could maximise tumour destruction. Our previous work (Griffin *et al.*, 1994) established a direct relationship between charge and volume regression in a mammary carcinoma. A clinical application of interest is the treatment of (multifocal) hepatic metastases from colorectal carcinomas. That treatment of lesions in the liver is feasible is suggested by the encouraging results of an uncontrolled trial for DCT of primary hepatoma in China (Lao *et al.*, 1994). In the approach towards clinical trial, we report here on the patterns of necrosis induced preclinically in liver itself, which will be the primary normal tissue at risk in this proposed new therapy for the condition.

### Materials and methods

All procedures to be described complied with the Animals (Scientific Procedures) Act 1986 (UK). Male adult outbred white (OBW) rats bred in the Paterson Institute were used throughout, at a weight of 230-250 g. Up to four rats were treated simultaneously while under general anaesthetic. Anaesthesia was induced by inhalation of enflurane in an ether chamber and then maintained by inhalation of halothane and oxygen via a facial mask system with scavenging of waste gases for the duration of the procedure. After induction of anaesthesia, the peritoneal cavity was opened by a small transverse subcostal incision through the skin and rectus muscle and the left lobe of liver exteriorised. Twin gold electrodes, 0.3 mm in diameter, were then inserted at 90° to the surface of the liver and a constant 10 mm apart within the central region of the exposed lobe. A dry gauze swab placed between the exteriorised liver and the underlying anterior abdominal wall of the animal acted as an insulator, preventing current return via any route other than through the substance of the liver between the two electrodes. The electrodes were held firmly in position by a supporting gantry above the animal. New gold electrodes were used after every 2-3 treatments, thus minimising the effects of gradual roughening of the electrode surface due to dissolution of gold at the anode. Before use, electrodes were sterilised with 70% alcohol. Direct current was then passed between the electrodes by means of a computer-controlled, constant-current power supply, which continually monitored voltage, which was normally in the range 1-16 V. In all procedures the electrode on the animal's left was the anode. In one series of experiments, current was held constant at either 1 or 5 mA and the duration of application of current was 10, 20, 30, 60 or 90 min. In a second series, treatment time was fixed at 30 min and current varied from 1 to 5 mA. Animals were randomly allocated to a current-time treatment group with 9-15 animals per group. After treatment, the liver was returned to the peritoneal cavity and the abdomen closed with 2/0 dexon suture. The animals were then returned to clean cages and allowed to recover from anaesthetic. At 48 h, a time at which necrosis had been found to be fully and consistently developed, treated livers were removed for examination under terminal inhalation anaesthesia. Estimation of the volume ($V$) of a cylinder of necrosis centred around the site of electrode placement was performed using vernier calipers to measure the diameter ($d$) of the visible necrotic area on the liver surface and the liver thickness ($t$) at that point. The equation

$$V = \pi d^2 t/4$$

was then applied. The treated liver was then placed in a Petri dish and covered with ice-cold buffered physiological saline solution before proton magnetic resonance (MR) imaging on a 4.7 tesla Biospec (Bruker/Oxford Instruments) MR system. Proton images were acquired using a 3-cm-diameter single-turn surface coil. A spin-echo pulse sequence was used, with a repetition time of 1.55 s and echo delay time of 40 ms,

Correspondence: JV Moore
Received 1 December 1994; revised 15 February 1995; accepted 17 February 1995

as described previously (Dodd *et al.*, 1993). After imaging, the liver was transferred to a histology cassette and placed in Bouin's solution for fixation before sectioning and staining with eosin and haematoxylin. Areas of necrosis were measured from (i) monochrome photographs of $^{1}$H-MR images of fresh *ex vivo* livers and (ii) histological slides of fixed material, using a Videoplan image analysis system (Kontron, Slough, UK).

Curve fitting and statistical analysis of experimental data were performed by means of a fitting programme (Biosoft, Cambridge, UK). Linear and higher degrees of regression were examined and the correlation coefficient obtained. Where regression analysis failed to provide a satisfactory fit (secondary damage *vs* primary damage), data were fitted to an asymmetric sigmoid equation.

### Results

Histological examination of treated livers clearly showed that the direct current induced two distinct patterns of necrosis. Firstly, a cylinder of tissue centred around each electrode underwent prompt colliquative necrosis with profound loss of histological architecture, extravasation of blood cells and intravascular thrombosis. This phenomenon was observed at each electrode, whether an anode or cathode, and in every animal treated. The cylinder of injury was, strictly speaking, elliptical; in measurements on enlarged photographs (23 livers) the plane *perpendicular* to the axis between the electrodes was slightly but significantly longer ($P<0.02$, paired $t$-test). In sharp contrast to this cylindrical 'central' necrosis at the electrode site (primary damage), a wedge-shaped region of pale coagulative necrosis, showing all the hallmarks of ischaemic injury, could be seen in many of the treated livers, with its apex at or around the site of electrode placement (i.e. within the zone of central necrosis) and its base extending to the liver edge. Proton magnetic resonance images of the treated liver also clearly show these two distinct regions of damage, at anode and cathode, both *in vivo* and *in vitro* (Figure 1).

Within 1 month of treatment, the regions of primary damage had been replaced by viable liver tissue associated with hyperplasia and the infarcts had resolved to a narrow fibrotic band. Analysis of the data obtained by treating the animals with (i) constant current for variable time and (ii) variable current for a fixed time showed no statistically significant difference ($t$-test) between the two methods, within the range examined. Consequently, the results from both experiments were pooled for subsequent data analysis. The volume of primary damage (or, in the two-dimensional images, area) was found to be consistently greater around the anode than around the cathode. Regression analysis of the data showed a linear relationship between volume of primary damage and charge passed (Figure 2). The lines of best fit are given by the equations:

Anode $V = (15.2 \pm 0.5)C - (9 \pm 5)$ $r = 0.96$

Cathode $V = (10.2 \pm 0.2)C - (2 \pm 5)$ $r = 0.92$

where $V$ represents the volume of necrosis (in mm$^3$) and $C$ represents the charge passed (in coulombs).

Likewise, regression analysis showed a linear relationship between area of primary damage ($A$, assessed from the two-dimensional MR images) and charge passed. The lines of best fit are given by:

Anode $A = (2.63 \pm 0.09)C + (1.41 \pm 0.86)$ $r = 0.92$

Cathode $A = (1.69 \pm 0.07)C + (0.8 \pm 0.6)$ $r = 0.90$

The relationship between area of secondary damage and charge passed was best represented by a sigmoidal function, for both anodic and cathodic damage (Figure 3). Again, the effect was greater at the anode than at the cathode and a distinct threshold level of charge appeared to be required in

**Figure 1** Proton magnetic resonance image of the left lateral lobe of rat liver, 2 days after DCT. 1, Cathodic primary damage; 2, cathodic infarct; 3, anodic primary damage; 4, anodic infarct; 5, patent radial blood vessels proximal to zones of DCT injury; 6, congested radial vessels distal to primary DCT damage. Vertical dimension of the overall liver lobe = 30 mm.

**Figure 2** Volume of DCT-induced primary necrosis at the anode (■) and the cathode (□), measured by calipers on freshly excised liver, as a function of electrical charge passed. The error bars represent one standard deviation for groups of nine animals.

**Figure 3** Area of DCT-induced secondary damage, measured from $^{1}$H-MR images of liver 2 days after treatment, as a function of electrical charge passed. The results are for (■) anode and (□) cathode. The error bars represent one standard deviation for groups of 15 animals.

order to produce any peripheral necrosis at the cathode. Comparison of primary and secondary damage showed a sigmoidal relationship, as would be expected. However, the area of secondary damage resulting from a given area of primary damage was statistically significantly greater at the anode.

Of particular interest was that the area of damage predicted by magnetic resonance imaging ($A_{MRI}$), which *in vivo* will be a non-invasive measurement, correlated with that obtained by histology ($A_{HIST}$). Using results for both anode and cathode:

Primary $A_{MRI} = (1.00 \pm 0.07)A_{HIST} - (1.76 \pm 1.66)$ $r = 0.96$

Infarct: $A_{MRI} = (0.63 \pm 0.05)A_{HIST} + (6.15 \pm 3.55)$ $r = 0.95$

The correlation was better for primary than secondary damage. In general, the extent of secondary injury for a given charge passed varied more widely than did primary damage. Nonetheless, the apparent trend towards lesser secondary 'damage' predicted by MR imaging than histology at 48 h remains unexplained at present; full time-course experiments are under way.

### Discussion

When low-level direct electrical current (units to tens of milliamperes range) is passed through living tissue, a number of pathological changes are seen. It has been known since the late nineteenth century that tissue destruction around the site of implanted electrodes takes place and that the magnitude of this destruction is greater at the anode than the cathode (Althaus, 1875). By the mid-nineteenth century, it had also been observed that such current could be used to promote thrombosis, particularly around the anode and, indeed, this technique gained some popularity as a means of treating aneurysms (Editorial, 1873) until superseded by advances in surgery later in the century. Although used successfully to treat uterine fibroid tumours in the late nineteenth century (Martin, 1886), interest in the mechanism of action and possible therapeutic use of low-level direct current waned until reawakened by a paper describing its use to treat a subcutaneous sarcoma in mice (Humphrey and Seal, 1959). Since that time, an increasing number of workers have published work describing the use of this modality against subcutaneous animal tumour models (Schauble *et al.*, 1977; David *et al.*, 1985; Marino *et al.*, 1986) and its potential clinical application (Nordenström, 1983). Our own research, using a mammary carcinoma growing subcutaneously in mice, revealed a linear relationship between the volume of regression induced in such a tumour and the charge passed through it, greater effect being observed when the intratumoral electrode was made an anode rather than a cathode (Griffin *et al.*, 1994).

Most recent preclinical work in this field has concentrated on the effects of direct current on tumours *per se*, but, like any proposed anti-cancer therapy, the utility of DCT will be determined by its *therapeutic* efficacy, i.e. the quantitative and qualitative relationship of injury in tumour to that in the involved normal tissue(s). As noted, our clinical interest is in metastases in liver, a well-structured normal tissue. We were particularly interested in the quantitative and qualitative aspects of any necrosis produced, in the relationship of such necrosis to implanted electrodes and in the possible mechanisms involved.

In common with other workers (Samuelsson *et al.*, 1980; Samuelsson and Jönsson, 1981) we found necrosis to occur at both anode and cathode, its magnitude directly proportional to the charge passed but with greater destruction around the anode than cathode (slopes of the lines being 15 and 10 $mm^3 C^{-1}$ respectively). A cylinder of necrosis could be seen around each electrode which on closer examination showed a slight elongation in a direction *perpendicular* to the plane joining the two electrodes. Important mechanistic conclusions can be drawn from this observation. David *et al.* (1985) suggested that electric current *per se* may have a direct effect on cells and their growth, while Miklavčič *et al.* (1993) observed that necrosis of a tumour mass can occur when two electrodes are placed on either side of the tumour. They concluded that the necrosis was directly due to the current flow between the electrodes, possibly by field effects on transmembrane potential and membrane permeability. Our investigations clearly show that, for liver, damage is localised around the electrodes with no distortion towards the region between anode and cathode, as would be expected if electric field density played a dominant role. In contrast, there was some (slight) elongation of the area of necrosis in the general direction of blood flow within the organ. This pattern of necrosis appears to be consistent with that resulting from diffusion of toxic products of electrolysis from each electrode. It is known that when direct current is passed the following electrochemical reactions take place:

At the anode:

$$3H_2O - 2e^- \rightarrow 2H_3O^+ + 1/2O_2$$

In addition the anodic dissolution of gold:

$$Au + Cl^- \rightarrow AuCl + e^-$$

$$Au + 4Cl^- \rightarrow AuCl_4- + 3e^-$$

At the cathode:

$$2H_2O + 2e^- \rightarrow H_2 + 2OH^-$$

Although we detected no differences between the biological effects of constant current for variable time and variable current for fixed time, it is possible that differences would have been observed if a wider range of current had been examined. If cell death is induced by toxic electrolytic products such as $H_3O^+$ and $OH^-$, their rate of production would be expected to influence their effectiveness, since at low current they might be 'neutralised' or harmlessly removed in the bloodstream. It has also been shown by others that the yield and distribution of electrolytic products is dependent on current density (Samuelsson and Jönsson, 1980). We do not believe the necrosis to result from thermal effects as others have found no evidence of heating at the same or higher power densities used by us (Samuelsson and Jönsson, 1981; David *et al.*, 1985; Heiberg *et al.*, 1991; Miklavčič *et al.*, 1993).

In addition to this central necrosis around the electrodes, direct current induced a quantitatively much more variable, but in the main more extensive, region of necrosis extending from the electrode site outwards to the liver edge. It is likely that this peripheral necrosis resulted from thrombosis within feeding vessels passing close to the electrodes (Figure 2). When the electrode was made an anode, infarcts could be seen from the lowest charge levels, but a threshold level of charge seemed necessary to induce such an infarct at the cathode. It has long been known that anodic current can be used to promote thrombosis in blood vessels; it seems likely from our observations that, in this tissue at least, thrombosis can be induced at and around the cathode, but less readily than at the positive electrode. The size of the infarct increases with the charge passed at both anode and cathode, once above the threshold. This behaviour seems logical as infarct size would depend on the number of end-arteries undergoing thrombosis, which would in turn be determined by the size of the region of central necrosis. As the primary necrosis increases, the area of infarct tends towards a plateau that is lower for the cathode than the anode. Thus it appears that the area of infarct is not wholly dependent on the size of the primary necrosis, but is influenced directly by the greater thrombogenic effect at the anode. We suggest that the current-induced ischaemic necrosis may be an important contributory factor in the 'field effect' reported by others.

These observations have important practical consequences if direct current therapy is to be used as a therapeutic modality in the treatment of malignant disease. It offers promise as a low-cost, minimally invasive way of destroying some primary or secondary tumours, but our work to date on neoplastic and normal tissue has shown that the modality can destroy both. It is important, therefore, that potential target tumours are carefully selected with consideration given to both optimum placement of treatment electrodes and potential damage to surrounding normal tissue. In sites where significant damage to surrounding normal tissue would be catastrophic (such as gastrointestinal tumours because of the risk of perforation) direct current therapy would probably not be appropriate, but in tissues where a degree of normal tissue necrosis would be acceptable (e.g. lung or liver), this modality may have a role to play. When used in such situations, electrode placement would be important, as in order to destroy tumours with minimal damage to normal tissue, it would be necessary to place electrodes close to vessels feeding the tumour and within the tumour itself (maximise primary and secondary damage within the tumour tissue). Angiography would, therefore, be desirable at the time of electrode placement.

**Acknowledgements**
DTG was funded as a Research Registrar through the generosity of the North West Regional Health Authority. NJFD and JVM are supported by the Cancer Research Campaign. We are grateful to Mr DA Broadbent for expert assistance.

**References**

ALTHAUS J. (1875). Further observations on the electrolytic dispersion of tumours. *Br. Med. J.* (Nov 15), 606–607.

DAVID SL, ABSOLOM DR, SMITH CR, GAMS J AND HERBERT MA. (1985). Effect of low-level direct current on *in vivo* tumour growth in hamsters. *Cancer Res.*, **45**, 5625–5631.

DODD NJF, MOORE JV, TAYLOR TV AND ZHAO S. (1993). Preliminary evaluation of low-level direct current therapy using magnetic resonance imaging and spectroscopy. *Phys. Med.*, **9**, 285–289.

EDITORIAL. (1873). The electropuncture treatment of aneurysm. *Br. Med. J.*, Dec 6, 667–668.

GRIFFIN DT, DODD NJF, MOORE JV, PULLAN BR AND TAYLOR TV. (1994). The effects of low-level direct current therapy on a preclinical mammary carcinoma: tumour regression and systemic biochemical sequelae. *Br. J. Cancer*, **69**, 875–878.

HEIBERG E, NALESNIK WJ AND JANNEY C. (1991). Effects of varying potential and electrolytic dosage in direct current treatment of tumours. *Acta Radiol.*, **32**, 174–177.

HUMPHREY CE AND SEAL EH. (1959). Biophysical approach towards tumour regression in mice. *Science*, **130**, 388–389.

LAO Y-H, GE T-E, ZHENG X-L, ZHANG J-Z, HUA Y-W, MAO S-M AND FENG X. (1994). Electrochemical therapy for intermediate and advanced liver cancer: a report of 50 cases. *Eur. J. Surg.*, Suppl. 574, 51–53.

MARINO AA, MORRIS D AND ARNOLD T. (1986). Electrical treatment of Lewis lung carcinoma in mice. *J. Surg. Res.*, **41**, 198–201.

MARTIN FH. (1886). Electrolysis in gynaecology; with a report of three cases of fibroid tumour successfully treated by the method. *J. Am. Med. Assoc.*, **7**, 61–68; 85–90.

MIKLAVČIČ D. SERŠA G, KRYŽANOWSKI M, NOVAKOVIČ S, BOBANOVIĆ F, GOLOUH R AND VODOVNIK L. (1993). Tumour treatment by direct electrical current-tumour temperature and pH, electrode material and configuration. *Bioelectrochem. Bioenerget.*, **30**, 209–220.

NORDENSTRÖM BEW. (1983). *Biologically-closed Electrical Circuits*, pp. 269–317. Nordic Medical Publications: Stockholm.

NORDENSTRÖM BEW. (1989). Electrochemical treatment of cancer. I. Variable response to anodic and cathodic fields. *Am. J. Clin. Oncol.*, **12**, 530–536.

SAMUELSSON L AND JÖNSSON L. (1980). Electrolytic destruction of lung tissue. Electrochemical aspects. *Acta Radiol. (Diagnosis)*, **21**, 711–714.

SAMUELSSON L AND JÖNSSON L. (1981). Electrolytic destruction of tissue in the normal lung of the pig. *Acta Radiol. (Diagnosis)*, **22**, 9–14.

SAMUELSSON L, OLIN T AND BERG N. (1980). Electrolytic destruction of lung tissue in the rabbit. *Acta Radiol. (Diagnosis)*, **21**, 447–454.

SCHAUBLE MK, HABAL MB AND GULLICK HD. (1977). Inhibition of experimental tumour growth in hamsters by small direct currents. *Arch. Pathol. Lab. Med.*, **101**, 294–297.

XIN Y-L. (1994). Advances in the treatment of malignant tumours by electrochemical therapy. *Eur. J. Surg.*, Suppl. 574, 31–36.